# Involvement of inflammation-related miR-155 and miR-146a in diabetic nephropathy: implications for glomerular endothelial injury

**DOI:** 10.1186/1471-2369-15-142

**Published:** 2014-09-02

**Authors:** Youqun Huang, Yan Liu, Ling Li, Baihai Su, Lichuan Yang, Wenxin Fan, Qinghua Yin, Lijia Chen, Tianlei Cui, Jie Zhang, Yanrong Lu, Jingqiu Cheng, Ping Fu, Fang Liu

**Affiliations:** 1 Division of Nephrology, West China Hospital of Sichuan University, Chengdu, Sichuan 610041, China; 2 Laboratory Animal Center of Sichuan University, Chengdu, Sichuan 610041, China; 3 Key Laboratory of Transplant Engineering and Immunology, Ministry of Health, Regenerative Medicine Research Center, West China Hospital of Sichuan University, Chengdu, Sichuan 610041, China

## Abstract

**Background:**

MicroRNAs have been demonstrated to play an important role in the pathogenesis of diabetic nephropathy (DN). In this study, we investigated both the repertoire of miRNAs in the kidneys of patients with DN and their potential regulatory role in inflammation-mediated glomerular endothelial injury.

**Methods:**

The miRNA expression profiling of the renal biopsy samples was performed by a microarray analysis; then, *in situ* hybridization and real-time polymerase chain reaction (PCR) were used to determine the localization and expression of two of the miRNAs significantly up-regulated in human DN kidney samples, miR-155 and miR-146a, in the kidney tissues from type 1 and type 2 DN rat models. Human renal glomerular endothelial cells (HRGECs) cultured under high-glucose conditions were transfected with miR-155 and miR-146a mimics, and the transforming growth factor (TGF)-β1, tumor necrosis factor (TNF)-α, and nuclear factor (NF)-κB expressions were examined by western blot, real-time PCR, and an electrophoresis mobility shift assay.

**Results:**

The expression of both miR-155 and miR-146a was increased more than fivefold in the kidney samples of the DN patients compared with the controls, and the miR-155 expression was closely correlated with the serum creatinine levels (R = 0.95, *P* = 0.004). During the induction and progression of the disease in type 1 and type 2 DN rat models, miR-155 and miR-146a were demonstrated to increase gradually. *In vitro*, high glucose induced the over-expression of miR-155 and miR-146a in the HRGECs, which, in turn, increased the TNF-α, TGF-β1, and NF-κB expression.

**Conclusions:**

Taken together, these findings indicate that the increased expression of miR-155 and miR-146a in the DN patients and in the experimental DN animal models was found to contribute to inflammation-mediated glomerular endothelial injury.

## Background

Complications affecting the macro- and microvasculature are major causes of illness and death among diabetic patients. For example, diabetic nephropathy (DN), one of the microvascular complications of diabetes mellitus, leads to the development of end-stage renal disease [1]. In recent years, our knowledge of DN pathophysiology has improved on both genetic and molecular levels. Although metabolic and hemodynamic alterations are traditionally considered to be responsible for renal injury in diabetes and DN is considered to be a non-immune disease, accumulating evidence now indicates that the immune system is involved in the development and progression of its pathophysiology [2]. DN is closely associated with the chronic low-grade inflammation and activation of the innate immune system [2-4]; and this is observed in patients with diabetes in the elevated serum levels of antibodies [5,6] and several inflammatory cytokines, primarily interleukin (IL)-1, IL-6, and IL-18, as well as C-reactive protein and tumor necrosis factor (TNF)-α [2,3,5].

MicroRNAs (miRNAs) are short non-coding RNAs that modulate physiological and pathological processes by inhibiting target gene expression through the blockade of protein translation or by inducing mRNA degradation. Recent findings have revealed critical functions for specific miRNAs in several cellular and biological processes, including proliferation, differentiation, and development, as well as in the regulation of genes relevant to cancer [6,7] and the modulation of immune responses [8,9]. Although several studies have evaluated the role of miRNAs in cancer, much less is known about their role in the field of diabetes and DN. Increasing evidence, predominantly from animal models of diabetes, shows that several fibrosis-related miRNAs, including miR-192 [10,11], miR-21 [12], miR-377 [13], and miR-221 [14], are involved in hyperglycemic conditions in different intrinsic renal cell types. Specific miRNAs, such as miR-155 and miR-146a, were initially linked with the inflammatory response by virtue of their potent up-regulation in multiple immune cell lineages by Toll-like receptor ligands, inflammatory cytokines, and specific antigens [15-17]. However, the pathogenic role of these miRNAs in the development of DN remains unknown.

Diabetic nephropathy primarily affects the glomerulus [18]. Glomerular endothelial cells (GEC) are strategically situated within the capillary loop adjacent to the glomerular mesangium and serve as targets of metabolic, inflammatory, biochemical, and hemodynamic signals that regulate the glomerular microcirculation [19]. However, the change in the GEC miRNA expression in response to hyperglycemic stimuli and the role of miRNAs during early and progressive DN are unclear.

In the present study, therefore, we aimed to clarify the repertoire of miRNAs in the development and progression of DN and the potential regulatory roles of miR-155 and miR-146a in endothelial injury. Furthermore, we investigated the mechanisms by which the nuclear factor (NF)-κB signaling pathway mediates the high glucose-induced expression of miR-155 and miR-146a in cultured human renal glomerular endothelial cells (HRGECs).

## Methods

### Human kidney sample preparation

Six patients with type 2 DN were included in this study (two women and four men, age 51.7 ± 7.8 years), and the kidney tissues were obtained from renal biopsies. Renal clinical staging and pathologic classification of these six patients were referred to Mogosen stage of DN [20] and pathologic classification of DN established by the Renal Pathology Society [21]. Three patients who were diagnosed with renal carcinoma and were undergoing nephrectomy but were shown to be free from hypertension, diabetes, and other co-morbidities were included as controls (one woman and two men, age 44 ± 12.9 years, urine test and serum creatinine were normal) (Table 1). The OGTT of the three controls included in our study was normal, thus, we thought they were not diabetes. Kidney tissues were obtained from renal biopsies (patients) or nephrectomy surgery (controls, primarily kidney cortexes) after receiving signed informed consent from the individuals. Tissues were used for a miRNA microarray and real-time polymerase chain reaction (PCR) or fixed in formalin and embedded in paraffin for histological and in situ hybridization. Changes in the renal morphology were examined in formalin, paraffin-embedded tissue sections (4 μm) stained with Periodic Acid-Schiff (PAS). The study was approved by the Ethics Committee on Human Research at Sichuan University (Sichuan, China).

**Table 1:** Demographics of patients with DN

	**DN**	**Control**
n	6	3
Age (years)	51.7 ± 7.8	46.5 ± 6.7
Sex (male)	5	2
Smoking (%)	28.6	33.3
Duration of diabetes (years)	7.3 ± 5.3	0
Duration of DN (months)	12.1 ± 11.5	0
Diabetic retinopathy (n)	2	0
HbA1c (%)	7.5 ± 0.8	6.3 ± 0.4
Hemoglobin (g/L)	117.4 ± 30.1	132.5 ± 10.5
Serum albumin (g/L)	31.4 ± 9.9	46.5 ± 4.7
Serum creatinine(umol/L)	133.0 ± 60.8	77.4 ± 12.6
eGFR (ml/min)	62.2 ± 21.3	108.5 ± 14.8
TG (mmol/L)	1.7 ± 1.2	2.1 ± 0.7
CHOL (mmol/L)	5.4 ± 1.3	5.8 ± 1.1
HDL (mmol/L)	1.5 ± 0.4	1.4 ± 0.5
LDL (mmol/L)	3.2 ± 1.1	3.3 ± 1.3
Urinary protein excretion (g/24 h)	3.5 ± 2.0	0.062 ± 0.013
Mogensen stage of DN (n) [20]		
Stage IV	5	0
Stage V	1	0
Pathologic classification of DN (n) [21]		
Class 2	2	0
Class 3	2	0
Class 4	2	0

Data are n or means ± SD. The eGFR was calculated using the modification of diet in renal disease formula.

### MicroRNA microarray and data analysis

**Figure 1:**
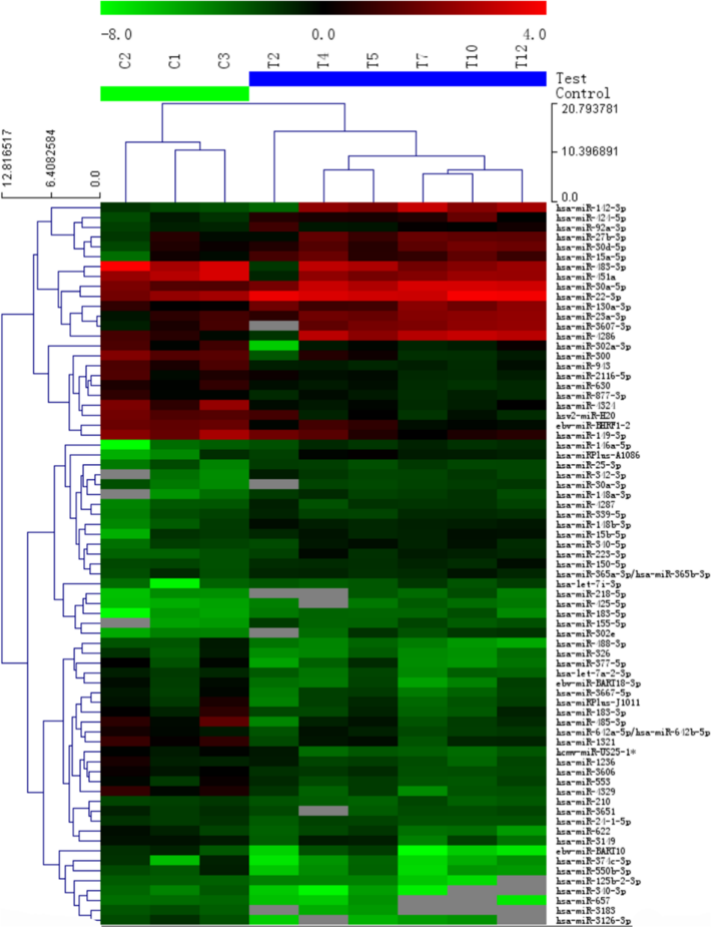
**Hierarchical clustering for the differentially expressed miRNAs in DN vs the controls.** The heat-map was generated by the unsupervised hierarchical clustering of the miRNA profiles of 6 diabetic patients and 3 healthy controls. The threshold we used to screen up- or down-regulated miRNAs was fold change ≥ 2.0. Heat map legend: Red = up-regulation, green = down-regulation. The gene and column rows represent the sample.

Dissected kidney tissue was homogenized, and RNA was extracted using TRIzol (Invitrogen, Carlsbad, CA, USA) according to the manufacturer’s protocol. The RNA was purified using a miRNeasy mini kit (QIAGEN) according to the manufacturer’s instructions. The RNA’s quality and quantity were determined using a Nanodrop 1000. To study the miRNA-expression in the diabetic patients, we performed a miRNA expression profiling of the kidney samples from six diabetic patients and three healthy controls using miRNA microarrays (Exiqon, Vedbaek, Denmark) according to the manufacturer’s instructions; the miRNA microarray service was provided by KangChen Bio-Tech, Inc., Shanghai, China. Briefly, after the RNA isolation, the miRCURY™ Hy3™/Hy5™ Power labeling kit (Exiqon, Vedbaek, Denmark) was used according to the manufacturer’s guidelines for miRNA labeling. One microgram of each sample was 3’-end-labeled with a Hy3TM fluorescent label by combining the RNA (one microgram) in 2.0 μL of water with 1.0 μL of CIP buffer (0.5 μL) and CIP (0.5 μL) (Exiqon). The mixture was incubated for 30 min at 37°C, and the reaction was terminated by incubation for 5 min at 95°C. Then, 3.0 μL of the labeling buffer, 1.5 μL of the fluorescent label (Hy3TM), 2.0 μL of the DMSO, and 2.0 μL of the labeling enzyme (this enzyme was actually a labeling enzyme from the miRCURYTM Array Power Labeling kit (Cat #208032-A, Exiqon)) were added to the mixture. The labeling reaction was incubated for 1 h at 16°C, and the reaction was stopped by incubation for 15 min at 65°C. The Hy3TM-labeled sample (25 μL) with 25 μL of hybridization buffer was denatured for 2 min at 95°C, incubated on ice for 2 min, and then hybridized to the miRCURYTM LNA Array (v.16.0) (Exiqon), according to the manufacturer’s instructions, for 16–20 h at 56°C in a 12-Bay hybridization system (Hybridization System; Nimblegen Systems, Inc., Madison, WI, USA). Following hybridization, the slides were washed several times using a wash buffer kit (Exiqon) and dried by centrifugation for 5 min at 400 rpm (the slides were dried by centrifugation for 5 min. at 1000 rpm). The slides were then scanned using the Axon GenePix 4000B microarray scanner (Axon Instruments, Foster City, CA, USA), and the images were imported using GenePix Pro 6.0 software (Axon Instruments) for the grid alignment and data extraction. The replicated miRNAs were averaged, and those with intensities ≥50 were selected to calculate the normalization factor. After normalization, the significant differentially expressed miRNAs were identified through Volcano Plot filtering. Hierarchical clustering was performed using MEV software (v4.6, TIGR) (Figure 1).

### In situ hybridization

Locked nucleic acid-based in situ detection of miRNA in kidney sections was performed. Briefly, the kidney samples were fixed at 4°C in formalin, embedded in paraffin wax, and cut into 4 μm sections. Dig-labeled probes were designed and synthesized by Invitrogen Inc. The sequences of the Dig-labeled probes against miR-155 and miR-146a mRNA were as follows: miR-155: 5′-CCCCTATCACGATTAGCATTAA-3′, miR-146a: 5′-AACCCATGGAATTCAGTTCTCA-3′. A scrambled probe sequence: 5′-TTCACAATGCGTTATCGGATGT-3′ was used as negative control for non-specific hybridization signal. In situ hybridization was performed using a detection kit on the sections of 4% paraformaldehyde-fixed (containing 0.1% diethylpyrocarbonate) kidney tissues according to the manufacturer’s instructions.

### Rat models

Littermate Sprague Dawley (SD) rats (male, aged 6 weeks, 200–220 g) were kept under laboratory standard conditions at a temperature of 22 ± 2°C, with a 12-h light/12-h dark cycle and relative humidity of 40%–60%. They were divided into two groups: the non-diabetic rats (controls, *n* = 6) and the streptozotocin (STZ)-induced type 1 diabetes rats (DM, *n* = 18). The DM rat models were induced by an intraperitoneal injection of a single dose of 60 mg/kg STZ (Sigma, St. Louis, MO, USA) in citrate buffer (pH 4.5). The random blood glucose level was tested using a glucometer (Accu-Chek, Roche, Basel, Switzerland), and blood glucose levels were determined on days 3 and 7 after the STZ injection. Only the rats with glucose concentrations ≥16.7 mmol/L for 3 consecutive days were used in the study. The DM rats were euthanized in weeks 1, 4, and 8 after the induction of diabetes, and their kidneys were harvested. Their renal cortexes were collected for real-time PCR by carefully removing the renal pelvis and medullar tissues. The experimental protocol was approved by the Animal Experimentation Ethics Committee at Sichuan University.

In a separate experiment to generate type 2 diabetes SD rats, rats (male, aged 6 weeks, 200–220 g) were allocated to one of two dietary regimens for an initial period of 6 weeks: a normal pellet diet (NPD, *n* = 6) or a high-fat and high-sugar diet (HFD, containing a regular diet plus 27.3% lard, 54.6% sucrose, 16.4% cholesterol, and 1.6% sodium cholate (w/w), *n* = 18). After this time, the HFD animals with high HOMA-IR (fasting plasma glucose (mmol/L) × fasting insulin (mIU/L) ÷ 22.5) were defined as insulin resistant and were injected with multiple low-doses of STZ (four doses of 25 mg/kg; Sigma, St. Louis, MO, USA) in citrate buffer (pH 4.5) after overnight fasting. The NPD animals were injected with a citrate buffer (1 mL/kg). The rats with fasting glucose levels ≥16.7 mmol/L 72 h after the STZ injection for 3 consecutive days were used for the study. The rats were kept on their respective diets until the end of the study. The DM rats were euthanized in weeks 0, 8, and 16 after the induction of diabetes, and their kidneys were harvested and their renal cortexes collected as before. All of the experimental protocols involving the animals were approved by the Animal Experimentation Ethics Committee at Sichuan University.

### Biochemical measurements in rat diabetes

Fasting (6 h) blood glucose levels were determined weekly for the first 2 weeks then every 2 weeks thereafter by using blood glucose meter (Optium™ Xceed Systems, Victoria, Australia). Before the disease induction (week 0), urine was collected for 24 h by placing rats in metabolic cages and performing a urinary albumin excretion assay. This process was repeated after the disease induction (for type 1 diabetes: weeks 1, 4, and 8; for type 2 diabetes: weeks 0, 8, and 16). The urinary albumin was measured by a competitive ELISA according to the manufacturer’s instructions (MaxiSorp Surface Immuno Plates™; Nunc, Roskilde, Denmark). The serum creatinine was detected by the QuantiChrom Creatinine Assay Kit (BioAssay Systems, Hayward, CA, USA).

### Quantitative real-time PCR for miR-155 and miR146a expression in the human and the rat kidneys

To confirm the miRNA profiling findings in the human subjects, we used a quantitative real-time PCR to measure the expression of miR-155 and miR-146a in both the human and the rat kidneys. The reverse transcription reaction used MMLV reverse transcriptase (Epicenter, Madison, WI, USA), and a quantitative PCR was performed using an ABI PRISM7500 system (Applied Biosystems, Foster City, CA, USA). miR-155 and miR-146a were further quantified with a TaqMan quantitative real-time PCR. The PCR primers used are shown in Table 2.

**Table 2:** The primer sequence of real-time PCR

**Gene**		**Primer sequences**
hsa-miR-155	RT	5′-GCGCGTGAGCAGGCTGGAGAAATTAACCACGCGCACCCCT-3′
	Forward	5′- CTTAATGCTAATCGTGAT -3′
	Reverse	5′- GAG CAG GCT GGA GAA -3′
hsa-miR-146a	RT	5′-GCGCGTGAGCAGGCTGGAGAAATTAACCACGCGCAACCCA -3′
	Forward	5′- CTGAGAACTGAATTCCA -3′
	Reverse	5′- GAG CAG GCT GGA GAA -3′
U6	RT	5′-CGCTTCACGAATTTGCGTGTCAT -3′
	Forward	5′- CTCGCTTCGGCAGCACATA -3′
	Reverse	5′- CGCTTCACGAATTTGCGTG -3′
TGF-β1	Forward	5′- CCAACTATTGCTTCAGCTCCA -3′
	Reverse	5′- GTGTCCAGGCTCCAAATGT -3′
TNF-α	Forward	5′- CCCAGGGACCTCTCTCTAATCA -3′
	Reverse	5′- AGCTGCCCCTCAGCTTGAG -3′
GAPDH	Forward	5′-CCCCTTCATTGACCTCAACTAC-3′
	Reverse	5′-GATGACAAGCTTCCCGTTCTC-3′

### Cell culture

Normal HRGECs were purchased from ScienCell Research Laboratories (Carlsbad, CA, USA) and cultured in an endothelial cell medium (ScienCell) containing 5% fetal bovine serum and 1% endothelial cell growth supplement at 37°C in a 5% CO_2_ atmosphere. The cells were serially passaged by a brief exposure to 0.25% trypsin (BD-Difco, USA) and 0.04% EDTA (Sigma, St. Louis, MO, USA), and the experiments were performed in the cells at passages 2–5 at 80% confluence. They were seeded at a density of 1 × 10^5^ cells/well in 6-well dishes or 2.5 × 10^5^ cells/25 cm^2^ flask. Prior to the experiments, the cells were fasted for 24 h in maintenance media.

To detect the time-dependent changes in the miR-155 and miR-146a expression, the cells cultured with mediums containing 25 mmol/L glucose were cultured at 37°C in 5% CO_2_ for 0, 0.5, 1, 2, 4, 8, and 24 h. They were then collected for a real-time PCR. Chemically modified RNA oligonucleotides comprising a sequence complementary to mature miR-155 and miR-146a (miR-155 and miR-146a inhibitors) were used to inhibit the miR-155 and miR-146a activities. The miR-155 and miR-146a mimics were double-stranded constructs consisting of a guide and passenger strands. An oligonucleotide containing a four-base mismatch (nontargeting scramble RNA) was used as a negative control. Pyrrolidine dithiocarbamate (PDTC, Sigma) was used as an NF-κB inhibitor. According to the set time points, the HRGECs (1 × 10^5^ per well) were transfected with miR-155 and miR-146a mimics, inhibitors, and scramble control after 24 h of starvation in a serum-free medium using Lipofectamine 2000 (Invitrogen) according to the manufacturer’s instructions.

### Western blot analysis

The proteins were extracted from the HRGECs using a RIPA lysis buffer and analyzed by western blot as described previously [17]. Briefly, after blocking the nonspecific binding with 5% bovine serum albumin, the membranes were incubated with primary antibodies against TNF-α and transforming growth factor (TGF)-β1 overnight at 4°C. After washing, the membranes were incubated with IRDye 800-conjugated secondary antibodies (Rockland Immunochemicals, Inc., Gilbertsville, PA, USA), and the signals were detected using the Odyssey Infrared Imaging System (Li-COR Biosciences, Lincoln, NE, USA) and quantitated with Image J software (NIH). The protein ratio was normalized against the GAPDH and expressed as the mean ± standard errors of the mean (SEM).

### Quantitative real-time PCR for mRNA expression in HRGECs

The total RNA was extracted from the HRGECs using the RNeasy kit according to the manufacturer’s instructions (Qiagen Inc., Valencia, CA, USA), and the cDNA was synthesized. A real-time PCR was performed with the Opticon®2 Real-Time PCR detector (Bio-Rad, Hercules, CA, USA) using the IQ SYBR green supermix reagent (Bio-Rad) as described previously [17]. The primers used to amplify the mRNAs of TNF-α, TGF-β1, and GAPDH are listed in Table 2. The housekeeping gene GAPDH was used as an internal standard. The ratios of specific mRNA:GAPDH mRNA were calculated and expressed as the mean ± SEM.

### EMSA of NF-κB in HRGECs

EMSA was performed using the LightShift™ chemiluminescent EMSA kit (Pierce Biotechnology, Rockford, Illinois, USA) according to the manufacturer’s protocol. Nuclear proteins were extracted from the HRGECs using a nuclear extraction kit (Pierce Biotechnology, Rockford, Illinois, USA) and incubated with a biotin-labeled NF-κB probe (5′-AGT TGA GGG GAC TTT CCC AGG C-3′) at room temperature for 20 min. Samples were separated by 6% non-denaturing polyacrylamide gel electrophoresis and transferred to a positively charged nylon membrane (0.45 μm, Pierce Biotechnology, Rockford, Illinois, USA).

### Statistical analyses

The data obtained were expressed as the mean ± SEM. The statistical analyses were performed using a linear correlation analysis and one-way analysis of variance followed by a two-tailed Newman-Keuls post-test (Prism 3.0 GraphPad Software, Inc., San Diego, CA, USA). *P*-values <0.05 were considered statistically significant.

## Results

### Characteristics of the research participants

The clinical and pathological characteristics of the patients are shown in Table 1. The cases of DN were notable for their statistically significant decreased estimated glomerular filtration rate (eGFR), increased proteinuria, serum creatinine levels, and increased glycosylated hemoglobin compared with the controls. They were also defined by the presence of diabetes, proteinuria, histological changes, and the absence of hepatitis, HIV, lupus, or other potential causes of glomerulonephritis. Three control samples were obtained from the healthy or biopsied samples of the unaffected portions of the tumor nephrectomies. The control subjects were defined as having normal serum creatinine levels and eGFR and an absence of proteinuria and hematuria.

### Aberrant expression of miR-155 and miR-146a between healthy and type 2 diabetic human kidney tissues

**Figure 2:**
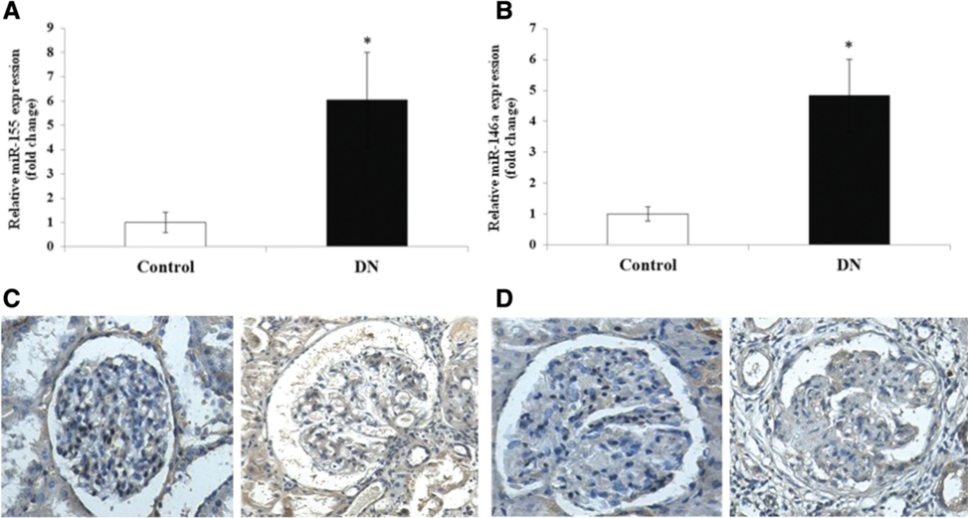
**The expressions and distribution of miR-155 and miR-146a in the kidneys from DN patients.** miR-155 and miR-146a were primarily localized in the glomerular endothelial cells, the mesangial areas and the tubular sections in kidneys from DN. **A**, **B**: miR-155 and miR-146a expressions in the kidney of the DN and controls were confirmed by quantitative real-time PCR. **C**, **D**: In situ hybridization was performed to determine the distribution of miR-155 and miR-146a in the kidneys from the DN patients. *p < 0.05.

The miRNA expression of the kidney tissues was analyzed using microarray screening. Compared with the normal controls, the 71 miRNAs in the type 2 DN patient samples were differentially expressed, with 32 elevated and 39 down-regulated (Figure 1). Thirty-one miRNAs were upregulated by ≥ 2-fold in the DN kidney samples from the DN patients, in which there were two significantly upregulated miRNAs, miR-155 (6.22 ± 2.81-fold) and miR-146a (4.87 ± 1.39-fold). These findings were further confirmed by a quantitative real-time PCR for miR-155 and miR-146a (Figure 2A,B).

The morphology of the kidney glomeruli from the DN patients was examined using PAS staining. The precise location and expression levels of the miR-155 and miR-146a were determined using *in situ* hybridization in the patient and control kidney tissue sections stained with Dig-labeled miR-155 and miR-146a riboprobes. The miR-155 and miR-146a were primarily localized in the GECs, mesangial areas and tubular sections of the DN kidneys, and the staining was significantly stronger in the DN GECs compared with the control samples (Figure 2C,D), which is consistent with the quantitative real-time PCR results above. To understand how these differentially expressed miRNAs might contribute to the inflammatory response, we searched for their potential regulatory targets using algorithms based on miRNA-mRNA complementarity and evolutionary conservation seed sequences (Target-Scan and PicTar). Surprisingly, some of the candidate targets of miR-155 and miR-146a were involved in the inflammatory responses (data not shown); therefore, we used miR-155 and miR-146a as targets for further study.

### Correlation between miRNA expression, serum creatinine levels, and urinary protein excretion

**Figure 3:**
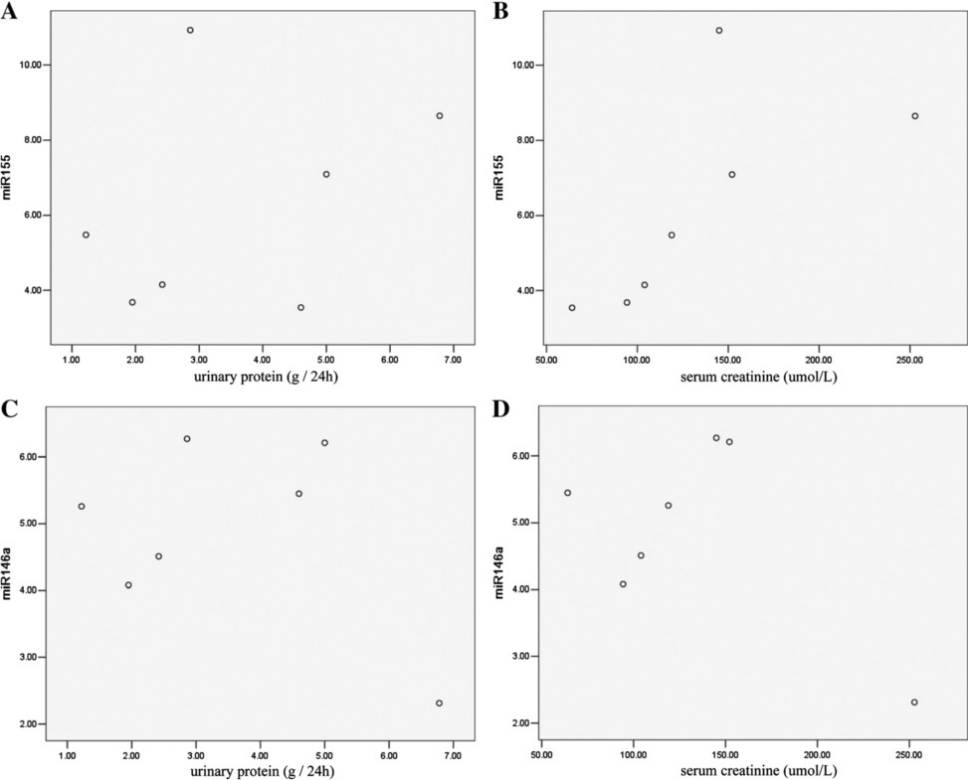
**Correlation of miR-155, miR-146a expressions and urinary protein excretion, serum creatinine of the DN patients.** Urinary protein was measured by a competitive ELISA and the serum creatinine level was detected by a QuantiChrom Creatinine Assay. The miR-155 and miR-146a expression levels were confirmed by a real-time PCR. A linear correlation analysis was performed between the miR-155 and urinary protein **(A)**; miR-155 and serum creatinine **(B)**; miR-146a and urinary protein **(C)**; and miR-146a and serum creatinine **(D)**. The linear correlation analysis showed the expression of miR-155 was closely correlated with the serum creatinine level (R = 0.95, P = 0.004).

The linear correlation analysis revealed that the miR-155 expression was closely correlated with the serum creatinine levels (R = 0.95, *P* = 0.004) but not with the urinary protein excretion levels (R = 0.336, *P* = 0.461); there were also no significant correlations between the miR-146a and serum creatinine levels (R = 0.531, *P* = 0.220) or the urinary protein excretion levels (R = 0.360, *P* = 0.427) (Figure 3).

### Altered miR-155 and miR-146a expression in type 1 and type 2 DN rat kidneys

**Figure 4:**
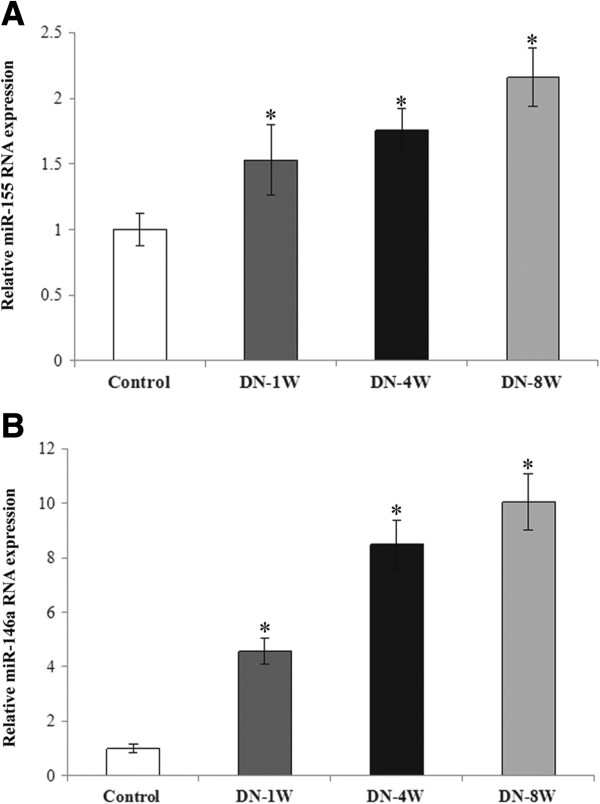
**Expression of miR-155 and miR146a in the STZ-induced rat kidney with type 1 diabetic nephropathy.** Quantitative real-time PCR was used to detect the expressions of miR-155 **(A)** and miR-146a **(B)** in the kidney of a STZ-induced Type 1 diabetic nephropathy model. Compared with the normal controls, the expressions of miR-155 and miR-146a were gradually increased from the time point of week 1 to week 8. *p < 0.05.

**Figure 5:**
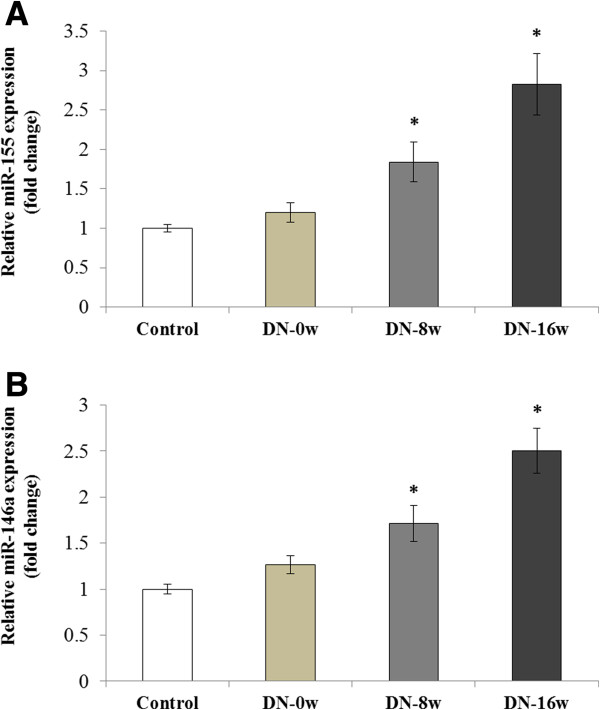
**Expression of miR-155 and miR146a in high-fat and high-sugar diet combined with STZ-induced rat kidney with type 2 diabetic nephropathy.** Quantitative real-time PCR was used to detect the expressions of miR-155 **(A)** and miR-146a **(B)** in the kidney of the Type 2 diabetic nephropathy model. Compared with the control group, the expressions of miR-155 and miR-146a were increased at the time points of week 8 and week 16. *p < 0.05.

In the rats with type 1 DN, the rats were sacrificed after the induction of diabetes at 1, 4, and 8 weeks (serum creatinine and urinary protein excretion were shown in Table 3), and a quantitative real-time PCR was used to show that the expression of miR-155 and miR-146a gradually increased during the progression of the DN (Figure 4A,B). A similar trend was observed for miR-155 and miR-146a expression in the development and progression of DN in the rats with type 2 DN (Figure 5A,B). In comparison with the NPD control group, the expression levels of miR-146a and miR-155 were significantly increased in the type 2 DN rats from week 0 to week 8 and 16 (Figure 5A,B) (serum creatinine and urinary protein excretion were shown in Table 4).

**Table 3:** Serum creatinine and urinary protein excretion in type 1 diabetes rat models

**Parameters**	**T1DM-week 0**	**T1DM-week 4**	**T1DM-week 8**
Serum creatinine (μmol/L)	33.9 ± 7.1	53.6 ± 11.2^a^	82.4 ± 20.0 ^ab^
urinary protein excretion (mg/24 h)	101.6 ± 9.8	227.8 ± 72.4^a^	296.9 ± 79.6 ^ab^

Note: ^a^*vs* week 0 (p < 0.05); ^b^*vs* week 4 (p < 0.05).

**Table 4:** Serum creatinine and urinary protein excretion in type 2 diabetes rat models

**Parameters**	**T2DM-week 0**	**T2DM-week 8**	**T2DM-week 16**
Serum creatinine (μmol/L)	28.6 ± 6.2	60.4 ± 6.9^a^	98.5 ± 24.7 ^ab^
Urinary protein excretion (mg/24 h)	130.5 ± 8.4	283.2 ± 69.5^a^	378.2 ± 85.9 ^ab^

Note: ^a^*vs* week 0 (p < 0.05); ^b^*vs* week 8 (p < 0.05).

### The role of the NFκB signaling pathway in miR-155 and miR-146a-mediated renal inflammation and fibrosis

**Figure 6:**
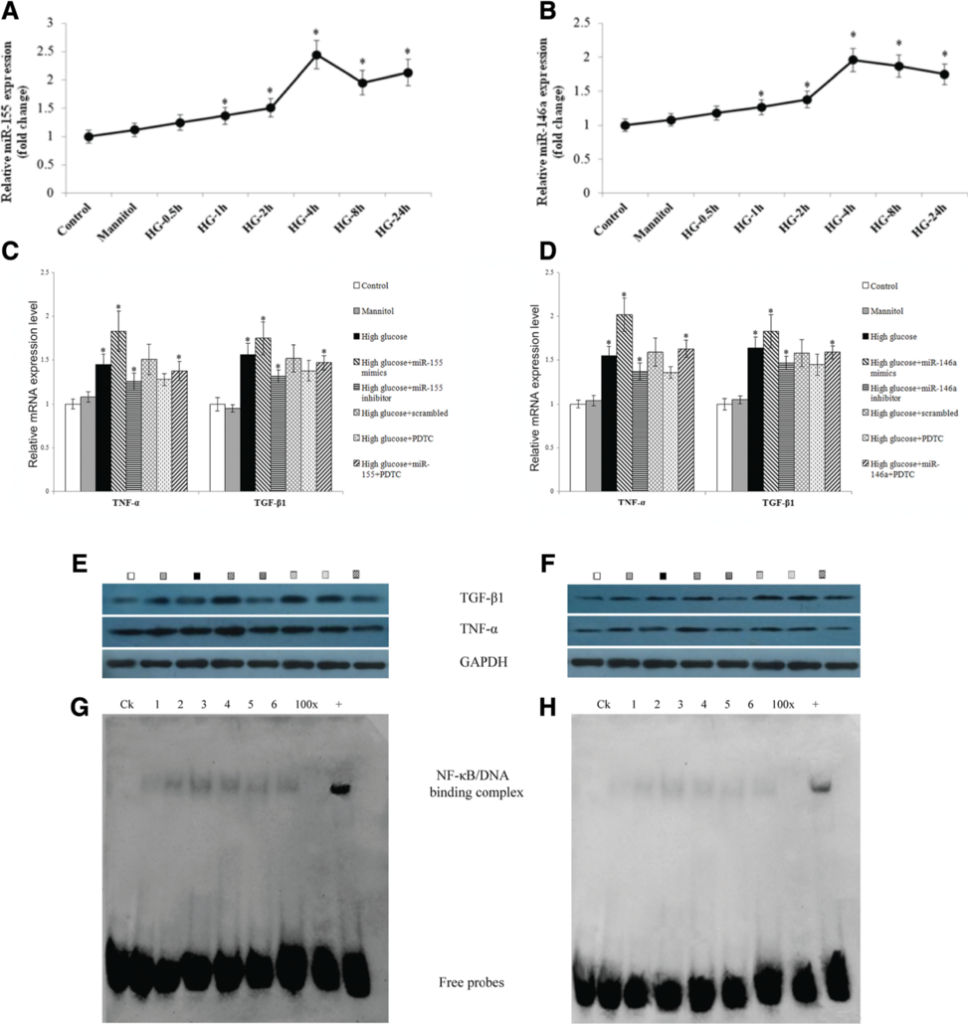
**Impact of miR-155 and miR-146a on HRGECs. A**, **B**. The expression of miR-155 and miR-146 under high glucose conditions of different times compared with the control and mannitol group; **C, D**. Quantification of miR-155 and miR-146a mRNA expression levels with different treating methods on HRGECs by a real-time PCR; **E**, **F**. Western blot was used to assess the protein levels of TNF-α and TGF-β with the same treating methods in C and D, glucose group is compared with mannitol group; the high glucose + miR155 inhibitor and high glucose + miR155 mimics are compared to high glucose + scrambled miRNA. High glucose + PDTC is compared with high glucose + miR-155 + PDTC group; **G**, **H**. Extracted nuclear proteins were incubated with a biotin-labeled NF-κB probe to detect the DNA-binding activity of NF-κB. Lane 1, normal control group; lane 2, high glucose (25 mmol/L); lane 3, high glucose + miR-155/146a mimics (50 nmol/L); lane 4, high glucose + miR-155/146a inhibitor (100 nmol/L); lane 5, high glucose + PDTC (100 umol/L); lane 6, high glucose + miR-155/146a (50 nmol/L) + PDTC (100 μmol/L). *p < 0.05.

To determine the effects of high glucose on the expression of miR-155 and miR-146a, the HRGECs stimulated with a medium containing 25 mmol/L glucose were cultured at 37°C in 5% CO_2_ for 0, 0.5, 1, 2, 4, 8, and 24 h. The expression of both the miR-155 and miR-146a was up-regulated 2 h after the glucose stimulation, peaked after 4 h, and then decreased until 24 h (with a minor increase of the miR-155 expression) relative to the control and the mannitol groups (Figure 6A,B).To explore the biological effect of miR-155 and miR-146a on the HRGECs under high-glucose conditions, the HRGECs were categorized as the normal control (containing 5 mmol/L glucose), mannitol (20 mmol/L mannitol), high glucose (containing 25 mmol/L glucose), high glucose + miR-155 mimic (50 nmol/L), high glucose + miR-155 inhibitor (100 nmol/L), high glucose + Scrambled miRNA (50 nmol/L), high glucose + PDTC (100 μmol/L), and high glucose + miR155 (50 nmol/L) + PDTC (100 μmol/L) groups. These cells were transfected with miR mimics or inhibitors as indicated, then harvested 24 h after transfection for the real-time PCR analysis and after 48 h for the western blot analysis. Compared with the normal control and mannitol groups, high glucose levels induced the up-regulation of both proinflammatory cytokines (TNF-α) and pro-fibrotic growth factors (TGF-β1), as shown by both the real-time PCR (Figure 6C,D) and western blotting (Figure 6E,F). Under high-glucose conditions, the HRGECs overexpression of miR-155 or miR-146a following the transfection with miR-155 or miR-146a mimics showed an additive effect on the TNF-α and TGF-β1 upregulation. However, the induction by glucose was clearly blunted by the miR-155 and miR-146a inhibitors, suggesting that miR-155 and miR-146a could significantly induce renal inflammation and fibrosis and contribute to HRGEC injury.To clarify the underlying NF-κB signaling pathway, the DNA-binding activity of NF-κB was assessed by EMSA using nuclear extracts from the HRGECs. A strong nuclear activation of NF-κB was observed in the cells from the miR-155 and miR-146a group (Figure 6G,H). Furthermore, the addition of PDTC partially repressed the up-regulated expression of TNF-α and TGF-β1 caused by the activation of NF-κB in the miR-155 and miR-146a over-expressing groups.

## Discussion

Recent evidence suggests that miRNAs can serve as important therapeutic targets in a wide range of human diseases [22] in which they can regulate the expression of key genes [23]. As such, some miRNAs have been suggested to have the potential to be novel diagnostic and therapeutic targets for diabetic complications [24]. Reports have shown that several miRNAs (miR-192, −194, −204, −215, and −216) are highly and quite exclusively expressed in the kidney [25], of which miR-192 was found to target SIP1, a major effector of the TGF-β signaling pathway [10]. Moreover, miR-377 is up-regulated in human and mouse mesangial cells exposed to high-glucose levels and can lead to increased fibronectin production in DN [13], while miR-216a regulates the collagen type I alpha 2 gene through mechanisms involving inhibition of the RNA binding protein Ybx1 [26]. Under hyperglycemic conditions, miR-221 is induced in the HUVECs, which triggers the inhibition of the c-kit and impairs the HUVEC migration. The MC phenotypic transition is crucial for DN progression, and miR-215 has been shown to function as a key endogenous gene-silencing factor that mediates the TGF-β1-induced MC activation and fibronectin expression via the CTNNBIP1/β-catenin pathway [27].

The identification of new and key regulatory miRNAs in DN would be of great importance, and the present study found that among the 32 miRNAs up-regulated in the DN patients, miR-155 and miR-146a were significantly increased and primarily distributed in the GECs, mesangial areas and tubular sections. Furthermore, miR-155 and miR-146a were gradually up-regulated during the development and progression of type 1 and type 2 DN, indicating that they could be activated in a rat DN model. We detected miR-155 expression was closely correlated with serum creatinine levels, Creatinine clearance was used to calculate estimated glomerular filtration rate (eGFR) for detection of kidney dysfunction. When in patients particularly with advanced kidney failure, creatinine clearance usually overestimates the GFR [28], therefore miR-155 may be a good marker for prediction the GFR of the DN. miR-146a expression was not correlated with serum creatinine levels or urinary protein excretion, this lack of correlation could reflect the limited sample size and consequent low statistical power, therefore, it needs our further investigation with more samples.

In a murine model of chronic kidney disease (CKD), the expression of miR-146a was elevated in B6.MRLc1 CKD mice and shown to increase with the development of CKD [29]. A histopathological analysis of glomerular and interstitial lesions revealed that the mRNA expression of inflammatory mediators was significantly higher in B6.MRLc1 than in C57BL/6 mice, which may have been induced by increased miR-146a [29], as observed in our *in situ* hybridization results. Our experiments confirmed that high glucose can increase the expression of miR-155 and miR-146a in a time-dependent manner. The high glucose-induced up-regulation of TNF-α and TGF-β1 was augmented by the overexpression of miR-155 and miR-146a, thereby increasing renal inflammation and fibrosis and enhancing diabetic GEC injury, which is mediated through the activation of the NF-κB signaling pathway.

Researchers found that miR-155 directly targets SMAD5 [30] and SMAD2 [31]. In DN, bone morphogenetic protein 7 (BMP-7) is down-regulated [32], and previous *in vitro* studies have shown that BMP-7-induced Smad5 reduces Smad2/3 signaling downstream of TGF-β [33,34]. This leads to the down-regulation of TGF-β-dependent profibrogenic events in cultured cells, thus increasing the expression levels of fibrosis-associated extracellular matrix proteins in glomeruli and the cortical interstitium [33]. Researchers found that TGF-β can lead to the phosphorylation of Smad2/Smad3, which associates with Smad4 as a heteromeric complex and translocates to the nucleus. This complex binds directly to Smad-binding elements and associates with a plethora of transcription factors, thus leading to the transcriptional induction or repression of a diverse array of genes. William Kong el al. found Smad4 can directly bind to the promoter of miR-155 and can be activated through the TGF-β/Smad pathway [35]. Therefore, miR-155 and TGF-β1 may form a positive regulation loop mediated by the SMAD signaling pathway.

In the monocytic leukemia cell line THP-1, Konstantin et al. [8] observed the induction of miR-146a in response to TNF-α. This was found to be mediated by NF-κB, as mutations in the NF-κB binding site of the miR-146a promoter impaired the TNF-α activation effect. Moreover, data have shown that miR-155 can be up-regulated by TNF-α through NF-κB [36]. Together with our findings from the present study, these results lead us to speculate that miR-155 and miR-146a form a regulatory loop in the TNF-α/NF-κB pathway during DN development.

## Conclusions

In conclusion, the present study provides direct biological evidence for the pathogenic importance of miR-155 and miR-146a in DN. Our results demonstrated that miR-155 and miR-146a were up-regulated in the DN patient and experimental animal models and served as a mediator of the glucose-induced TNF-α/TGF-β1-NF-κB pathway. Taken together, these findings suggest that miR-155 and miR-146a are not only biomarkers but also mediators in the development of DN. Determination of the detailed miR-155 and miR-146a mechanisms that cause renal damage in the setting of diabetes could elucidate the pathogenesis of DN and enable improved treatment strategies to be developed.

### Consent

Written informed consents were obtained from the patients for publishing their medical data and any accompanying images. The copies of the written consents are available for review by the editor of this journal.

## Competing interests

The authors declare that they have no competing interests.

## Authors’ contributions

FL and PF participated in the design of the study, drafted the manuscript and contributed to the revision of the manuscript. YL carried out ELISA and the molecular genetic studies, helped to drat the manuscript and participated in the revision of the manuscript. YQH performed cell culture and human sample research. SBH, YLC and TLC participated in human sample preparation. WXF participated in the design of the study and performed the statistical analysis. LL, LJC, QHY, JZ contributed to the clinical and basic research. YRL and JQC participated in the design of the study. All of the authors participated in the discussions about the manuscript and approved the final version.

## Pre-publication history

The pre-publication history for this paper can be accessed here:

http://www.biomedcentral.com/1471-2369/15/142/prepub
